# Draft Genome Sequence of Sinorhizobium meliloti AK555

**DOI:** 10.1128/MRA.01567-18

**Published:** 2019-01-10

**Authors:** Victoria S. Muntyan, Olga A. Baturina, Alexey M. Afonin, Maria E. Cherkasova, Yuri V. Laktionov, Alla S. Saksaganskaya, Marsel R. Kabilov, Marina L. Roumiantseva

**Affiliations:** a All-Russian Research Institute for Agricultural Microbiology (ARRIAM), Laboratory of Genetics and Selection of Microorganisms, Saint Petersburg, Russia; b Institute of Chemical Biology and Fundamental Medicine, Siberian Branch of the Russian Academy of Sciences (ICBFM SB RAS), Novosibirsk, Russia; Queens College

## Abstract

The inoculation of legume seeds with Sinorhizobium bacteria significantly improves pasture production. Here, we report the draft genome sequence of symbiotically efficient and salt-tolerant Sinorhizobium meliloti inoculant strain AK555, which substantially increases biomass yield of a number of Medicago sativa subsp. varia varieties, such as “Agniya,” “Vega 87,” and “Selena.”

## Abstract (precis)

The inoculation of legume seeds with Sinorhizobium bacteria significantly improves pasture production. Here, we report the draft genome sequence of symbiotically efficient and salt-tolerant Sinorhizobium meliloti inoculant strain AK555, which substantially increases biomass yield of a number of Medicago sativa subsp.

## ANNOUNCEMENT

Sinorhizobium meliloti AK555 (RCAM00051, A3) was isolated from nodules of wild-growing Medicago falcata in northwest Kazakhstan in 2002 as a strain tolerant to 0.75 М NaCl (1–3). It forms a highly effective symbiosis with Medicago sativa subsp. varia var. “Agniya,” “Vega 87,” and “Selena,” according to data produced by Geographical Experiment Network with biologicals from 2009 to 2011 (4).

A culture of AK555 was started from a single colony grown in tryptone yeast (TY) medium (28°C, 20 h, 180 rpm) (5). Cells were harvested by centrifugation at 4,000 × *g* at an optical density at 600 nm (OD_600_) of >0.75. DNA was extracted with a FastDNA kit according to the instructions of MP Biomedicals and quantified with a spectrophotometer (Biophotometer; Eppendorf AG, Germany).

Genomic DNA was fragmented to an average size of 600 bp with the S2 instrument (Covaris) in a microTUBE AFA fiber snap-cap tube. The paired-end library was constructed using dual-index NEBNext multiplex oligos and an NEBNext Ultra II DNA library prep kit for Illumina (New England BioLabs [NEB]). The AK555 DNA library was sequenced with a reagent kit v3 (2 × 300 bp) on a MiSeq benchtop sequencer (Illumina) in a genomics core facility (ICBFM SB RAS), with a yield of about 1.4 million paired-end (PE) reads. The reads were quality trimmed, and adapter sequences were removed with the BBDuk tool from the BBMap package (ktrim=r k=23 mink=11 hdist=1 tpe tbo minlen=25 qtrim=rl trimq=10) with default parameters (6).

A MinION (Oxford Nanopore) sequencer R9.4 installed at the All-Russia Research Institute for Agricultural Microbiology (ARRIAM) was used to generate long reads. We constructed the barcoded DNA library with the 1D native barcoding genomic DNA protocol (with kits EXP-NBD103 and SQK-LSK108). We basecalled the raw fast5 files with Albacore version 2.3.1 with default parameters. The run yielded 1.6 million reads after basecalling (4.2 Gbp). We demultiplexed the resulting reads using Deepbiner version 0.2.0 (7) and cleaned the reads using Porechop version 0.2.3 (https://github.com/rrwick/Porechop), both with default parameters. In total, 26,889 reads with an *N*_50_ value of 31,266 bp comprising 195 Mbp were produced for the strain AK555.

Illumina and nanopore reads were assembled into 7 contigs by Unicycler version 0.4.6 (8) with the chosen “conservative” mode. The NCBI Prokaryotic Genome Annotation Pipeline (9) was applied for AK555 automatic functional genome annotation and resulted in 7,153 protein-coding genes, 3 rRNA operons, 54 tRNA genes, and 2 transfer-messenger RNAs (tmRNAs).

The genome of AK555 comprises a chromosome (SMc, circle of 3,675,317 bp), two circular megaplasmids (SMa, 1,658,858 bp, and SMb, 1,328,877 bp), and two cryptic plasmids (SMd, circle of 31,192 bp, and SMe, assembled from 3 contigs [440,009, 790, and 787 bp]). Genomic islands (GIs) as phage-related sequences with low G+C contents similar to those in strain Rm1021 (10) were not detected in the chromosome of AK555. Potential GIs represented by 41-kbp (G+C content, 58.67%; 91 open reading frames [ORFs]) and 51.4-kbp (G+C content, 59.7%; 108 ORFs) sequences integrated in tRNA-Gln (CTG) and in tRNA-Lys (CTT) with phage-related integrases and LigD (2 and 1, respectively) genes nearby.

### Data availability.

The genome sequence of Sinorhizobium meliloti AK555 was deposited in GenBank under the accession number PZMI00000000. The raw sequencing data are registered in the NCBI SRA database under accession number SRS3949116. This announcement describes the second version of the genome assembly.
